# The incidence of surgical site infection and its predictors among women delivered via cesarean sections in Ethiopia: a systematic review and meta-analysis

**DOI:** 10.3389/fmed.2024.1395158

**Published:** 2024-04-25

**Authors:** Temesgen Gebeyehu Wondmeneh, Jemal Abdu Mohammed

**Affiliations:** Department of Public Health, College of Medical and Health Science, Samara University, Semera, Ethiopia

**Keywords:** surgical site, infection, women, cesarean-section, Ethiopia

## Abstract

**Background:**

Although surgical wound infection remains a serious issue worldwide, the disease burden is greater in developing countries, including Ethiopia. Even though there were primary studies conducted at district levels in Ethiopia, there is little evidence about the pooled incidence of surgical site infections at the national level. Thus, this systematic review and meta-analysis determined the pooled incidence of surgical site infection and its associated factors among cesarean-delivered women in Ethiopia.

**Methods:**

We searched PubMed, CINAHL, African Journals Online, Google Scholar, and higher educational institutional repositories. A random-effects model was used to estimate the pooled effect size with 95% confidence intervals (CIs). Funnel plot and egger tests were computed to determine the existence of publication bias. A subgroup analysis was carried out.

**Results:**

Twenty-three studies were included in the final analysis. The pooled incidence of surgical site infection among women delivered via cesarean section was 12.32% (95% CI: 8.96–16.11%). Rural residence (AOR = 2.51, 95% CI: 1.15–3.87), membrane rupture (AOR = 2.04, 95% CI: 1.24–2.85), chorioammionitis (AOR = 4.13, 95% CI: 1.45–6.8), general anesthesia (AOR = 1.99, 95% CI: 1.22–2.75), post-operative Hgb level less than 11 mg/dL (AOR = 3.25, 95% CI: 1.54–4.96) and membrane rupture greater or equal to 12 h (AOR = 3.93, 95% CI: 1.93–5.92) were independent risk factors for surgical site infections.

**Conclusion:**

More than one in 10 women delivered via cesarean section developed surgical site infections in Ethiopia. Women living in rural areas and those with a membrane rupture, chorioammionitis, or anemia should be given special attention. General anesthesia should not be a mandatory procedure.

## Background

Surgical site infection continues to be an important cause of postoperative morbidity (1). It represents a major burden of disease for patients and health services (2). Most surgical wound infections are acquired from the patient’s own microbial flora in the operating room, while the remains are obtained mainly from operating room staff during surgery (3). Globally, the incidence of surgical site infections after a cesarean section was 5.63% (4). The incidence rate of surgical site infection was 0.15% in China (5), 8.02% in India (6), and 12.6% in Nepal (7). In the United Arab Emirates, surgical site infection was detected in 1.4% of the women who underwent cesarean operations (8). According to a WHO study with a special focus on surgical site infection in low and middle-income countries, the pooled prevalence of surgical site infection was 11.2 per 100 surgical patients (9). In Africa, the pooled prevalence of surgical site infection after cesarean section was 10.21% (10). In a study of Sub-Saharan Africa, the incidence of surgical site infection was 7.3% (11). After cesarean sections, 3.48% of women developed surgical site infections in Rwanda (12). In Ethiopia, the estimated prevalence of surgical site infections was 10.4% (13), 8.81% (14), and 9.72% (15). Risk factors for surgical site infection were older patients, more than 24 h of preoperative hospital stay, longer-duration procedures, emergency surgeries (16), and blood transfusion (17). Premature membrane rupture, diabetes, and hypertension during pregnancy, as well as a prolonged labor, were also other risk factors for surgical site infection after a Cesarean section (18, 19). Additionally, the risk of surgical site infection was raised by female genital tract infections (20), urinary tract infections in pregnancy, postpartum hemorrhage (21), chorioamnionitis, anemia (22), and a lack of antibiotic prophylaxis (23).

Studying the incidence of surgical site infection after cesarean section helps determine the current new infection and disease burden in developing countries like Ethiopia. Therefore, to develop preventive strategies, identifying the pooled incidence of surgical site infection after a cesarean section and its risk factors in Ethiopia is essential.

## Methods

### Protocol and registration

This systematic review and meta-analysis followed the Preferred Reporting Items for Systematic Reviews and Meta-Analyses (PRISMA) guidelines (24) (S1 File). This protocol has been registered in PROSPERO with ID CRD42022329641.

### Search strategies

Comprehensive searches were conducted using the databases of PubMed, CINAHL, Google Scholar, and African Journals Online to find potentially relevant articles. In addition, accessible Ethiopian higher education institutional repositories and the cross-reference lists of already identified articles were also systematically searched to obtain similar articles. Electronic database searches were initially conducted on April 10–18, 2023, and then updated on February 27–29, 2024. The key MeSH terms used for the PubMed database searches were “surgical wound infection,” “caesarean section,” and “Ethiopia.” For PubMed’s entry terms, “all fields” is used. For CINHA, Google Scholar, and African Journals Online, the same key words were used with “all fields.” For institutional repositories, we used the phrase “Surgical site infection among cesarean section women in Ethiopia.” See more comprehensive search strategies in the S2 File.

### Selection of included studies

The search strategy was implemented by two authors (TGW and JAM). Studies identified through different database searches were combined and exported using Endnote X8.1 software. Duplicated studies were removed with Endnote X8.1 software. TGW and JAM independently assessed the selected articles for their applicability to the review objective using their titles and abstracts. After the initial screening, the full texts of all studies considered relevant were obtained. Two reviewers (TGW and JAM) separately examined the eligibility of the full texts. Disagreement between the two reviewers (TGW and JAM) reached by scientific consensus.

### Outcome measurements

Incidence is a measure of risk that is the probability that a subject within a population will develop a given disease or other health outcome over a specified follow-up period. It can be calculated by dividing the number of subjects developing the disease over a certain period by the total number of subjects followed over that period (25). In this study, incidence is a measure of the risk of the probability that women delivered via cesarean section developed a surgical site infection over a specific follow-up period. Two parameters were required to estimate the incidence of surgical site infection: the number of cases who develop surgical site infection over a specific follow-up period and the total number of women delivered via cesarean section in the same period of time. The incidence was calculated by dividing the number of patients who developed surgical site infections over a certain period by the total number of women who undergo cesarean sections (sample size) over that period.

### Criteria for considering studies for the review

#### Inclusion criteria

All studies that reported the magnitude of surgical site infection (SSI) as a percentage among cesarean section women, as well as studies that provided the total number of women delivered via cesarean sections (N) along with the number of cases of surgical site infection (n) were included. Only studies written in English were included. There were no limitations on publication type, publication year, or study designs in this systematic review and meta-analysis. Studies conducted in Ethiopia were included.

#### Exclusion criteria

Any studies that could not be accessed at the time of the search process were excluded after at least two email contacts with correspondence authors because it is impossible to evaluate the quality of the studies in the absence of their full texts. Studies that failed to report surgical site infections as well as studies on systematic reviews and meta-analyses were excluded. Studies with low quality were not considered in this systematic review and meta-analysis. Studies conducted on a very select group of immune-compromised patients, such as HIV patients, were excluded since they could not be generalized to the general population because they were more vulnerable to infection. Case reports, case series, letters, opinions, notes, editorials, and conference abstracts were excluded.

### Data extraction

Using a standard data extraction format, the two authors independently extracted the necessary information from each study. The disagreement between two data extractors was resolved by scientific consensus. The primary author, publication year, region, study design, sample size, number of women with surgical site infection, and percentages of surgical site were retrieved for each included study (S3 File).

### Quality assessment

The methodological quality of the included studies was assessed by two independent authors (TGW and JAM). Any disagreement between the two authors was settled by scientific consensus. The quality of each included cross-sectional and cohort study was assessed by the Joanna Briggs Institute’s quality appraisal checklist (26). Studies that scored at least 50% were considered low-risk and included in the final meta-analysis.

### Statistical analysis

All the necessary data was retrieved from the articles using a Microsoft Excel spreadsheet and imported to STATA software version 15 for further analysis (27). A meta-analysis using the random-effects DerSimonian and Laird models was used due to expected heterogeneity (28). The random-effects model was employed when heterogeneity was greater than 50% (based on the I^2^ statistic) (29). Subgroup and sensitivity analyses were performed to find potential heterogeneity moderators when significant heterogeneity existed. Publication bias was checked by objectively computing the Egger test (30) and subjectivity inspecting funnel plots (31). The trim-and-fill method was applied when publication bias existed (32). The estimated pooled incidence of surgical site infection was presented by forest plots with a 95% CI. The pooled odds ratio was computed to determine factors associated with surgical site infection.

## Results

### Search results

**Figure 1:**
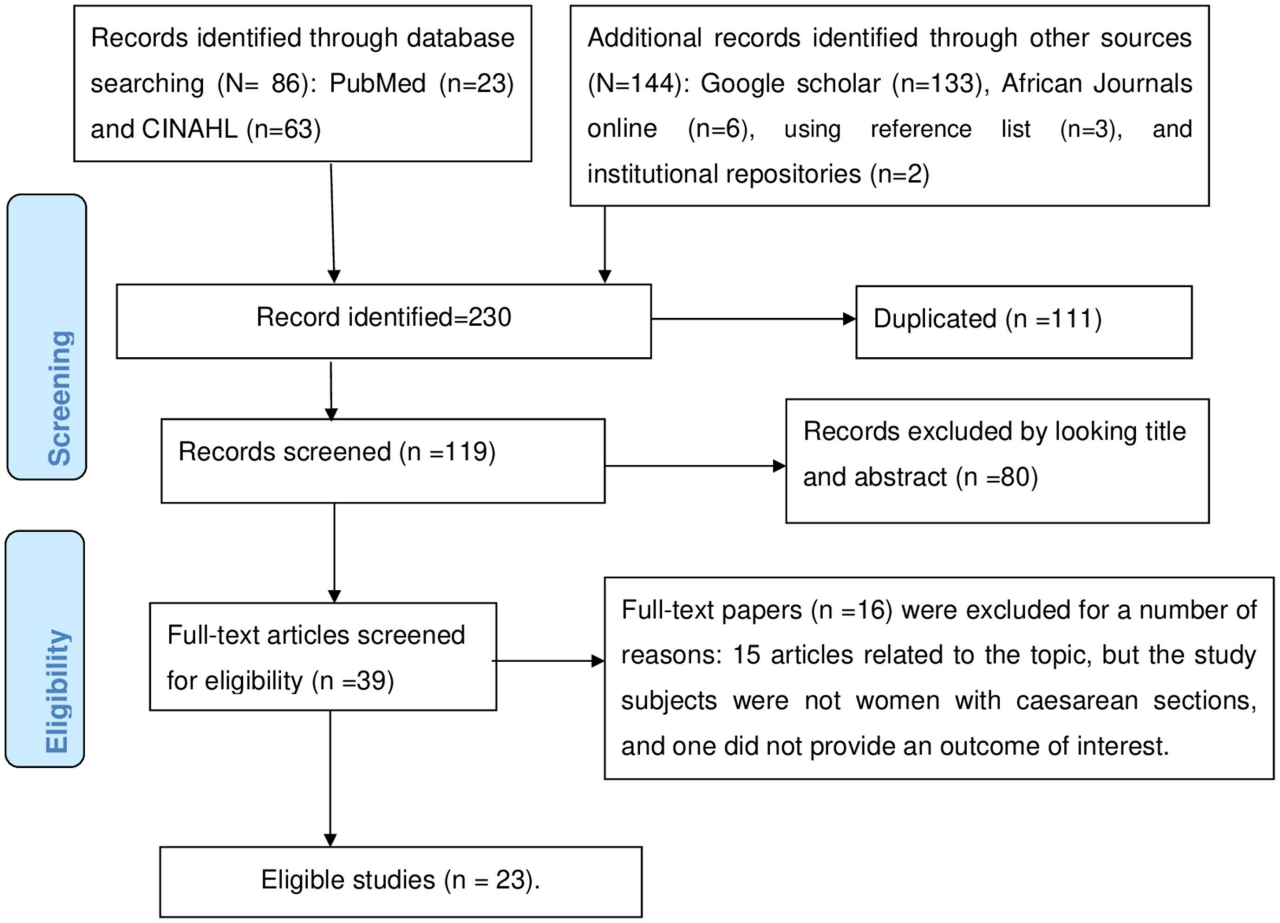
This shows the PRISMA flow chart for the selection of studies for systematic review and meta-analysis.

In the first search, 230 articles related to surgical site infections were retrieved via electronic databases. One hundred eleven were removed due to duplication, while 80 articles were excluded after reading their titles and abstracts due to their irrelevance. The remaining 39 articles were subjected to a full text review, 16 of which were removed because 15 studies failed to include women who had undergone cesarean sections as study participants, and one did not report an outcome of interest. Twenty three eligible studies were included in the final analysis. The specific screening procedures are depicted in a PRISMA flow chart (Figure 1).

### Characteristics of included primary studies

The final analysis included 23 studies with 10,201 participants; of them, 1,281 developed surgical site infections. Seventeen studies were cross-sectional, and six were cohort studies. The sample sizes of the studies ranged from 166 (33) to 1,069 (34). In the southern nation nationality people (SNNP) (35) and Amhara region (36), the highest (79.2%) and the lowest (2.4%) surgical site infection were reported, respectively. There were nine studies conducted in the Amhara region and four in the SNNP region. Addis Ababa and Oromia each had three studies, while two were from Tigray region and the other two from Harari region. All of the included studies had a low risk of bias (Table 1).

**Table 1:** Characteristics of included primary studies.

Authors	Publication year	Region	Study design	Sample size	Cases (%)	Risk of bias
Adane et al. (37)	2022	Harari	cohort	336	26 (7.74%)	Low
Alemye et al. (34)	2021	Harari	cross-section	1,069	131 (12.3%)	Low
Ali (33)	2017	Amhara	cross-section	166	12 (7.2%)	Low
Ali et al. (38)	2021	Amhara	cross-section	818	100 (12.2%)	Low
Amenu et al. (39)	2011	Oromia	cohort	580	66 (11.4%)	Low
Ayala et al. (40)	2021	Oromia	cross-section	382	34 (8.9%)	Low
Azeze et al. (41)	2019	Amhara	cross-section	383	30 (7.8%)	Low
Bizuayew et al. (42)	2021	Amhara	cross-section	622	77 (12.4%)	Low
Dach et al. (43)	2018	SNNP	cross-section	325	42 (12.9%)	Low
Gashaw et al. (44)	2022	SNNP	cross-section	431	51 (11.8%)	Low
Gedefaw et al. (45)	2018	Amhara	cross-section	447	42 (9.4%)	Low
Gelaw et al. (46)	2017	Tigray	cross-section	384	26 (6.8%)	Low
Gelaw et al. (47)	2018	A.A	cross-section	474	40 (8.4%)	Low
Kebede (35)	2022	SNNP	cross-section	226	179 (79.2%)	Low
Ketema et al. (48)	2020	Amhara	cohort	520	132 (25.4%)	Low
Lijaemiro et al. (49)	2020	A.A	cohort	166	25 (15%)	Low
Molla et al. (50)	2019	Amhara	cross-section	334	27 (8.1%)	Low
Rose et al. (51)	2018	Amhara	cohort	247	21 (8.6%)	Low
Wendmagegn et al. (52)	2018	Tigray	cross-section	206	24 (11.7%)	Low
Negese et al. (36)	2023	Amhara	cross-section	368	9 (2.4%)	Low
Mezemir et al. (53)	2023	A.A	cohort	741	86 (11.6%)	Low
Wodajo et al. (54)	2017	SNNP	cross-section	592	65 (11%)	Low
Mamo et al. (55)	2017	Oromia	cross-section	384	36 (9.4%)	Low

SNNP, southern nation nationality people, A.A, Addis Ababa.

### Quality of included studies

The quality of each cross-sectional and cohort study was independently evaluated by the two authors (TGW and JAM). The quality score of each included primary study, based on the JBI quality appraisal criteria, showed low risk for all studies. For cross-sectional and cohort studies, the quality score ranges from 6 to 8 and 8 to 11, respectively. The quality assessment of the generally agreed-upon conclusions from the primary studies that were included is displayed in S4 File.

### Pooled incidence of surgical site infections among women delivered via cesarean section

**Figure 2:**
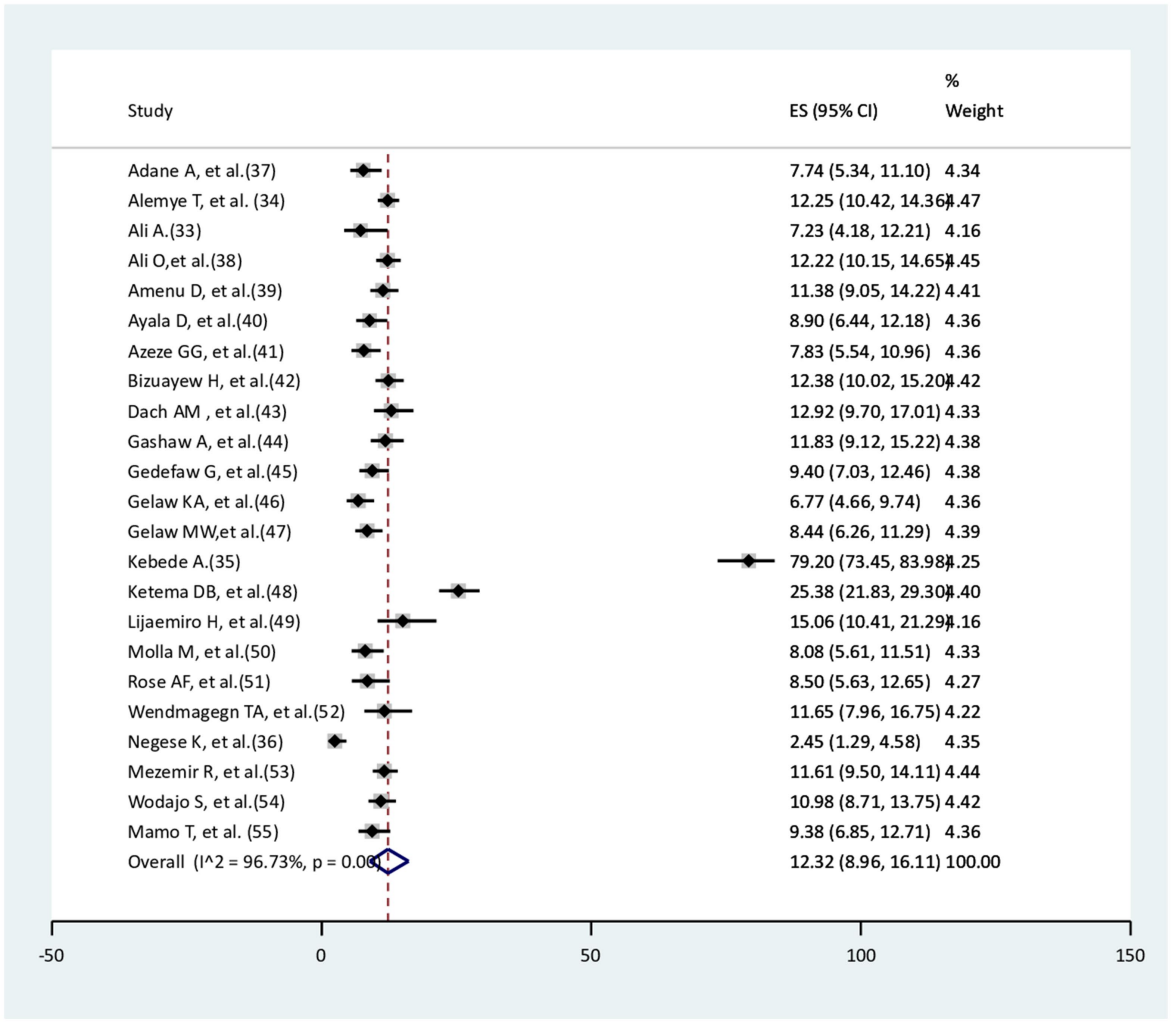
Pooled incidence of surgical site infection among cesarean section women.

The pooled incidence of surgical site infection among women delivered via cesarean section was 12.32% (95% CI: 8.96–16.11). There was a significant amount of heterogeneity between studies (I^2^ = 96.73%, *p* < 0.001; Figure 2).

### A sub-group analysis of the incidence of surgical site infection among women delivered via cesarean deliveries

Due to the significant heterogeneity across the included studies, a sub-group analysis based on study region, study design, publication year, and sample size was taken into consideration to identify a possible source of heterogeneity. The incidence of surgical site infection was highest in the southern nation nationality of people (SNNP) at 26.2% (95%CI: 5.4–55%), followed by Addis Ababa at 11.14% (95%CI: 8.1–14.6%), and Harari at 11.1% (95%CI: 9–13%). The highest heterogeneities (I^2^), which were 99.3% (*p* < 0.001) and 94.14% (p < 0.001), were observed in the southern nation nationality of people (SNNP) and Amhara region, respectively. The remaining regions lacked heterogeneity. There were comparable incidences of surgical site infection in the cohort and cross-sectional studies, with significant heterogeneity. In addition, in the sample size of less than or equal to 420 and in the sample size of greater than 422, comparable magnitudes of surgical site infection were noted. In the publication years 2020 and after, surgical site infection was 16% (95%CI: 9.2–24.28%) with significant heterogeneity (I^2^ = 98.48%, *p* < 0.001); in the publication year prior to 2020, it was 9.4% (95%CI: 8.4–10.5%), with moderate heterogeneity (Table 2).

**Table 2:** Sub-group analysis of the incidence of surgical site infection among women delivered via cesarean section.

Variables	Categories	Included study	Sample size	Estimated	Heterogeneity
				Incidence (95%CI)	I^2^ (%), *p*-value
Region	Amhara	9	3,905	9.8% (6.2–14.1%)	94.14, *p* < 0.001
	SNNP	4	1,574	26.2% (5.4–55%)	99.3, *p* < 0.001
	Oromia	3	1,346	10.1% (8.5–11.8%)	-
	Addis Ababa	3	1,381	11.14% (8.1–14.6%)	-
	Harari	2	1,405	11.1% (9–13%)	-
	Tigray	2	590	8.3% (6.2–10.7%)	-
Study design	Cross-sectional	17	7,611	12.2% (8–17.1%)	99.3, *p* < 0.001
	Cohort	6	2,590	12.8% (8.3–18.3%)	92.9, *p* < 0.001
Sample size	≤ 420	13	3,907	12.3% (6–20.6%)	97.99, *p* < 0.001
	> 420	10	6,294	12.4% (10.1–14.8%)	87.7, *p* < 0.001
Based on publication year	< 2020	12	4,522	9.4% (8.4–10.5%)	31.94, *p* = 0.14
	≥ 2020	11	5,679	16% (9.2–24.28%)	98.36, *p* < 0.001

Dash (−) indicates no heterogeneity, SNNP: Southern Nation Nationality People.

### Publication bias

**Figure 3:**
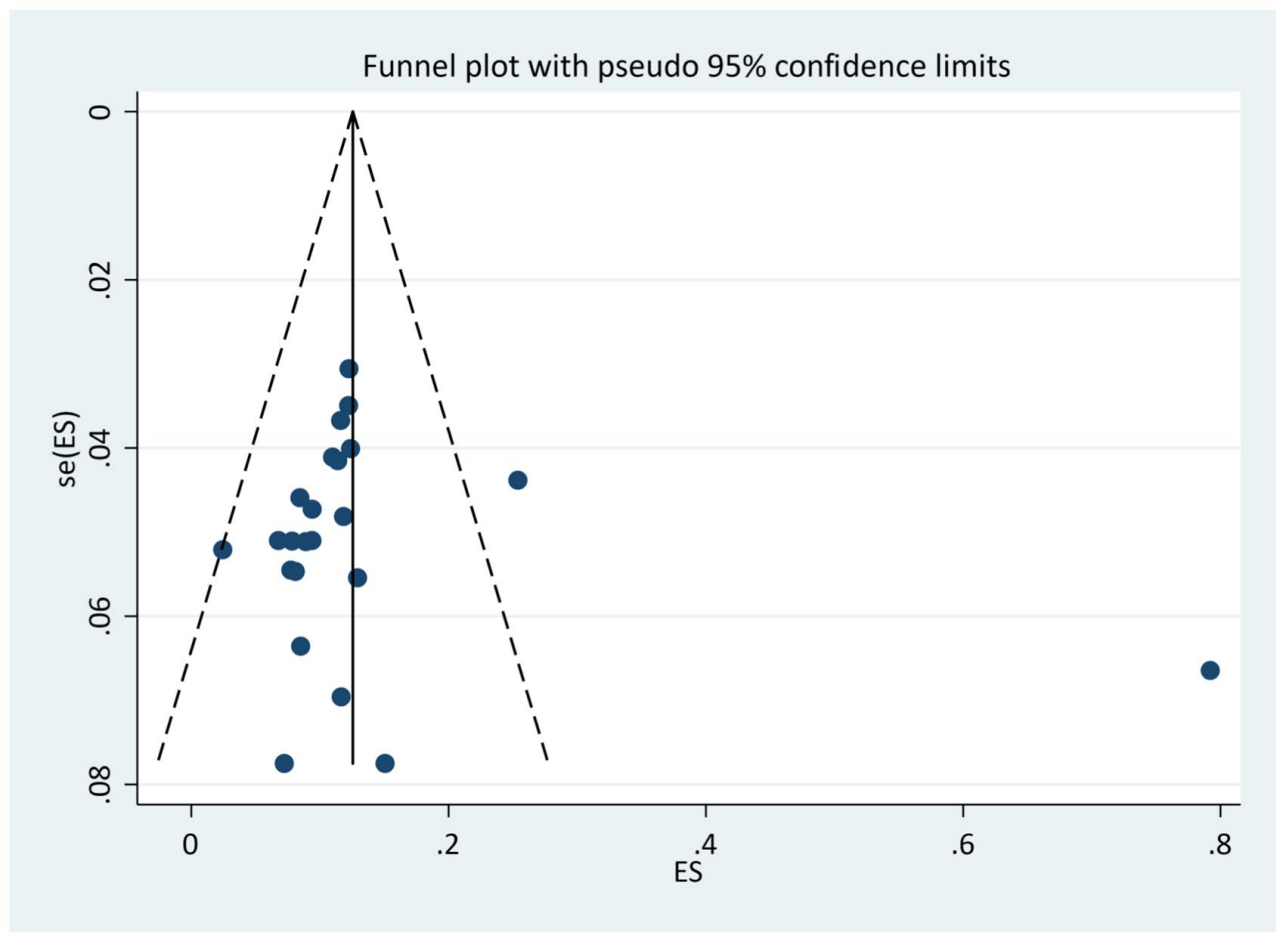
A funnel plot of publication bias for the incidence of surgical site infection.

In this systematic review and meta-analysis, the funnel plot indicated the asymmetric distribution of the studies (Figure 3), but the Egger’s test revealed no statistical significance [bias =1.61 (95% CI: −0.2.84–6.1), *p* = 0.46].

### Sensitivity analysis

**Figure 4:**
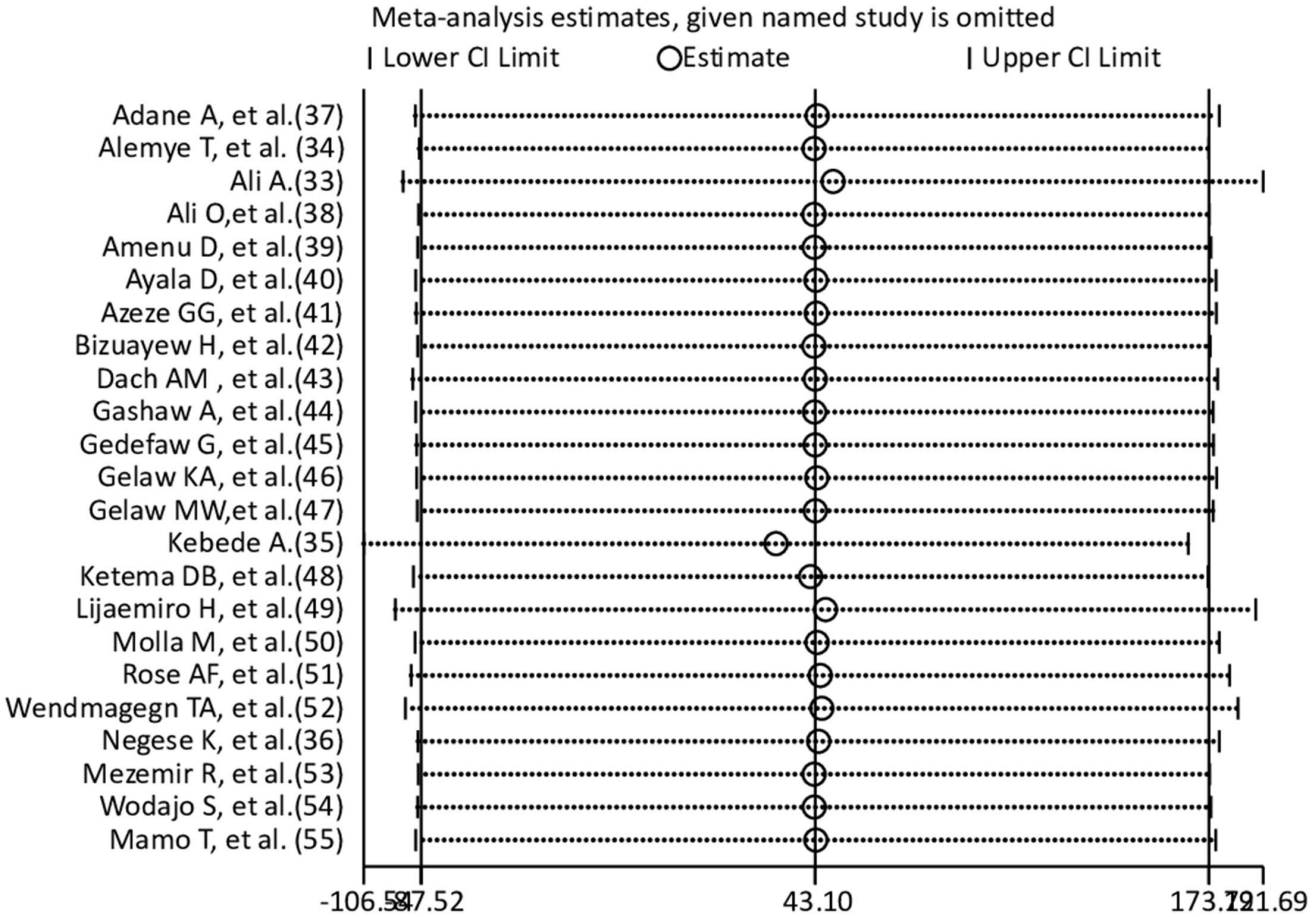
Sensitivity analysis for the incidence of surgical site infection.

A sensitivity analysis was carried out to see the effect of an individual study on the pooled effect size. According to the leave-one-out sensitivity analysis (Figure 4), there were no visible differences. The results of the meta-analysis showed it was stable.

### Risk factors associated with surgical site infections in cesarean section mothers

Place of residence, general anesthesia, rupture of the membrane, chorioammionitis, and a post-operative Hgb level less than 11 g/dL were risk factors for surgical site infection, but emergency cesarean section, hypertension, interrupted skin closure, and midline incision did not have a significant association with surgical site infection.

### The association between residence and surgical site infection

**Figure 5:**
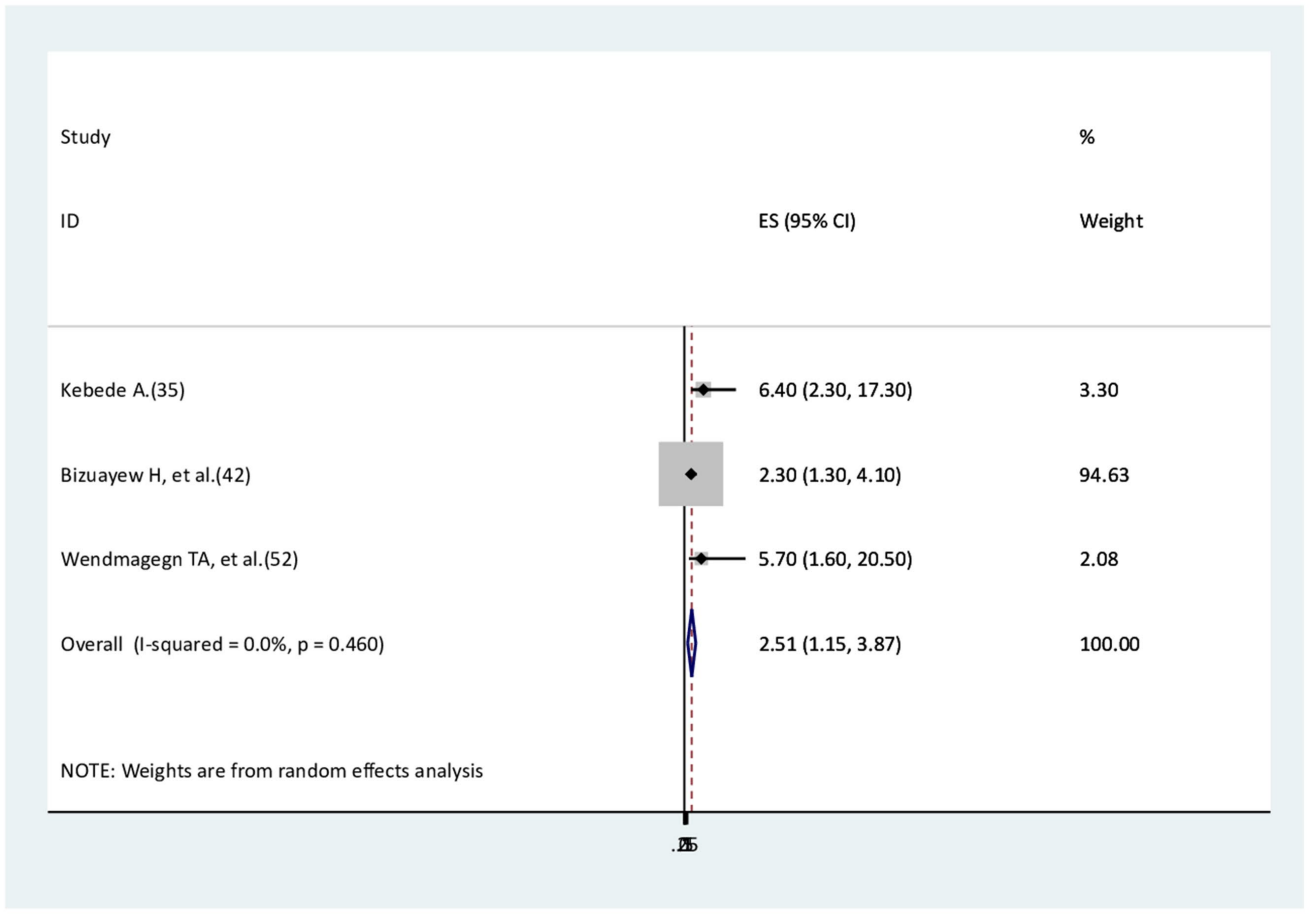
The association between residence and surgical site infection.

Three studies (35, 42, 52) were identified to check the association between rural residence and surgical site infections. All three studies found that rural residence is a risk factor for surgical site infection compared to urban residence. The findings of this meta-analysis revealed that rural residents were 2.5 times more likely to develop surgical site infections compared to urban residents (AOR = 2.51, 95% CI: 1.15–3.87). No evidence of heterogeneity was reported (I^2^ = 0.0%, *p* = 0.46; Figure 5).

### The association between hypertension and surgical site infection

**Figure 6:**
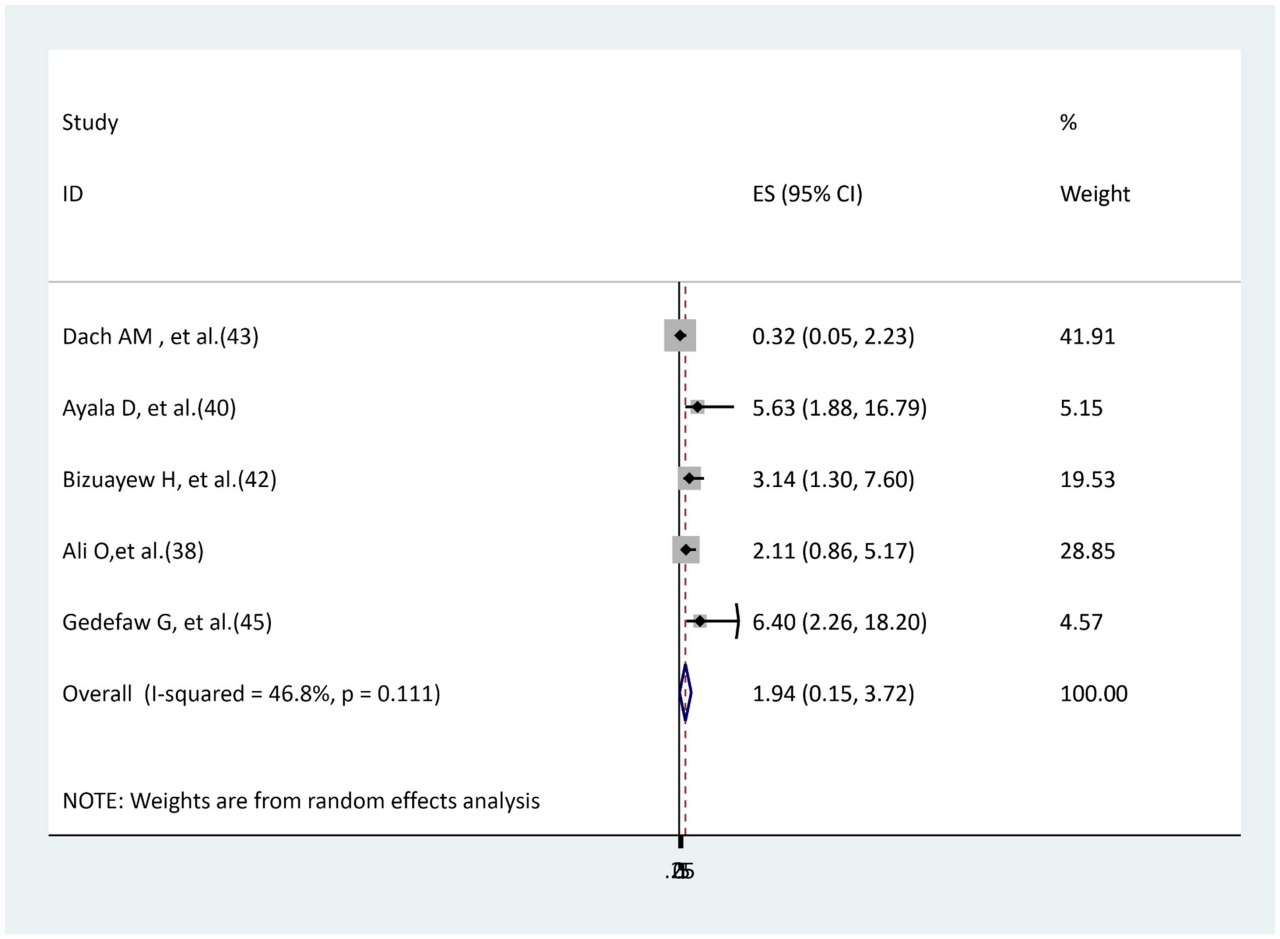
The association between hypertension and surgical site infection.

Five studies (38, 40, 42, 43, 45) were used to examine the relationship between hypertension and surgical site infection. Three of these primary studies (40, 42, 45) revealed a significant association, while the other two studies (38, 43) reported an insignificant association. The result of the random effect model meta-analysis showed that there is no significant association between hypertension and surgical site infection (AOR = 1.94, 95% CI: 0.15–3.72). Moderately insignificant heterogeneity was found (I^2^ = 46.8%, *p* = 0.111; Figure 6).

### The association between chorioammionitis and surgical site infection

**Figure 7:**
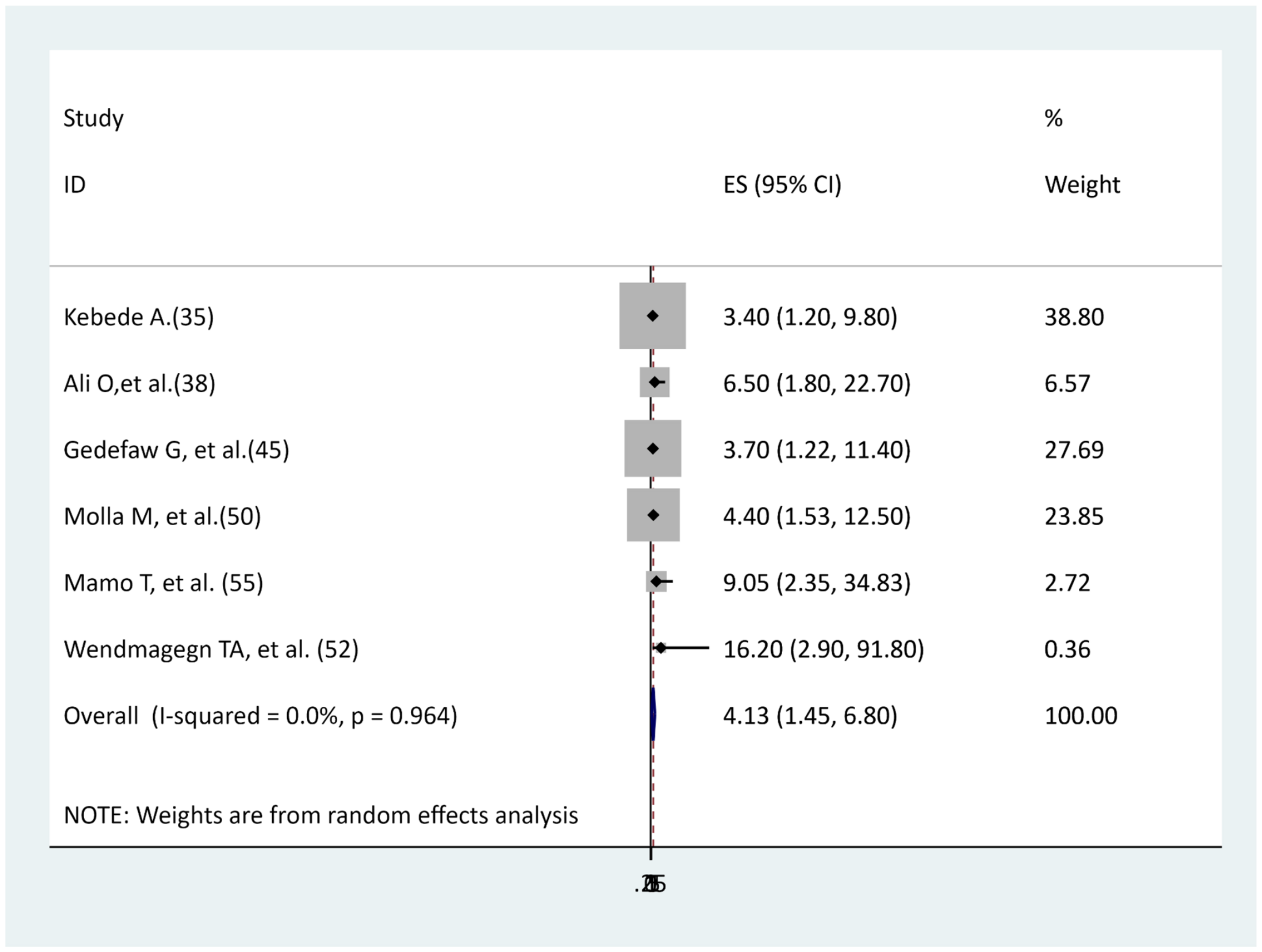
The association between chorioammionitis and surgical site infection.

Six studies (35, 38, 45, 50, 52, 55) were found to determine the association between chorioammionitis and surgical site infection, and all of the studies’ findings reported a statistically significant association. In the current meta-analysis, women with chorioammionitis during cesarean section were 4 times more likely to develop surgical site infection than women without chorioammionitis (AOR = 4.13, 95% CI: 1.45–6.8), and there was no evidence of heterogeneity (I^2^ = 0, *p* = 0.964; Figure 7).

### The association between emergency cesarean sections and surgical site infection

**Figure 8:**
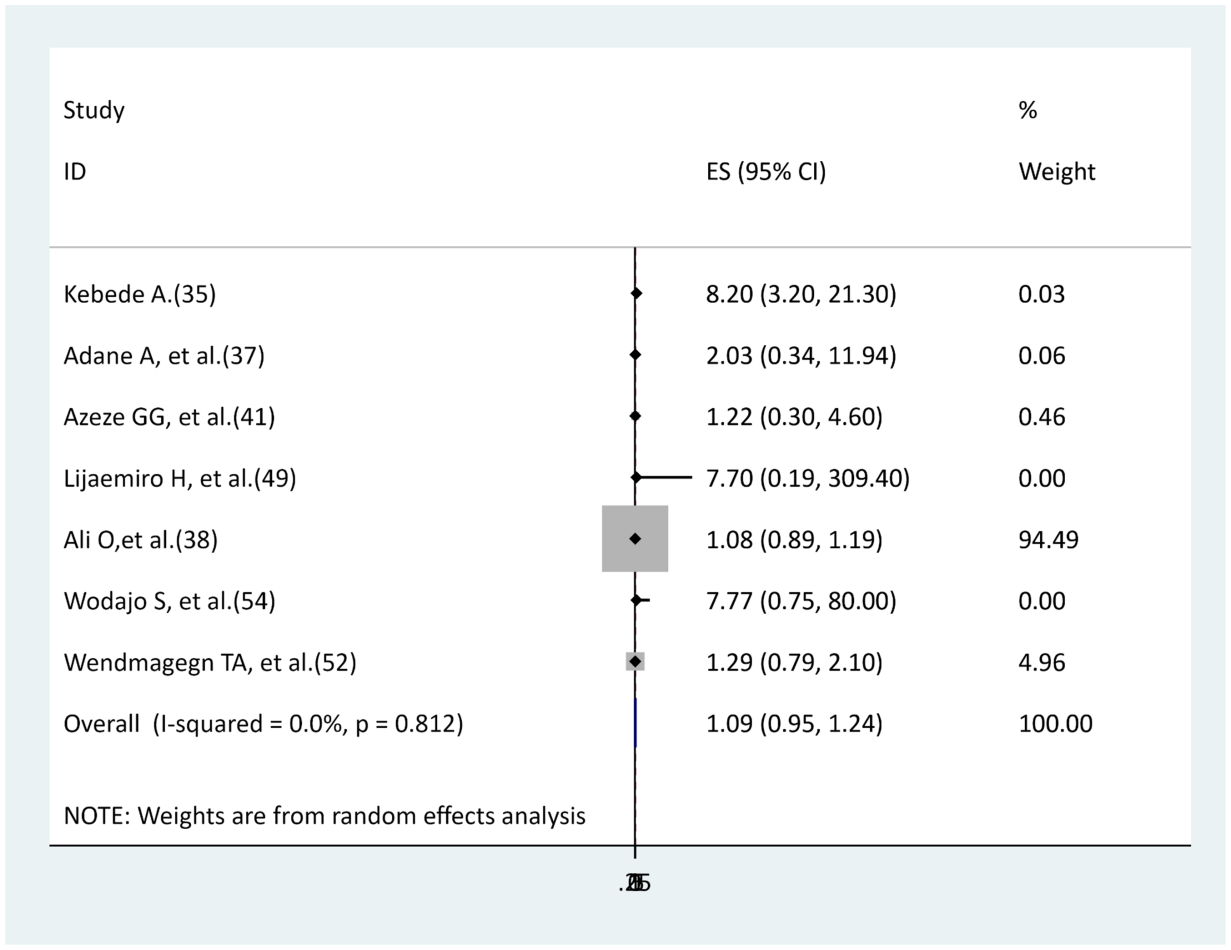
The association between emergency cesarean sections and surgical site infection.

Six studies (35, 37, 38, 41, 49, 52, 54) were included to determine the relationship between emergency cesarean sections and surgical site infections. Except for the one study (35) that found a significant association, the remaining five studies (37, 38, 41, 49, 52, 54) did not find a significant association. This meta-analysis revealed that no relationship existed between emergency cesarean section and surgical site infection (AOR = 1.09.95% CI: 0.95–1.24), with no heterogeneity (I^2^ = 0, *p* = 0.721; Figure 8).

### The association between membrane rupture and surgical site infection

**Figure 9:**
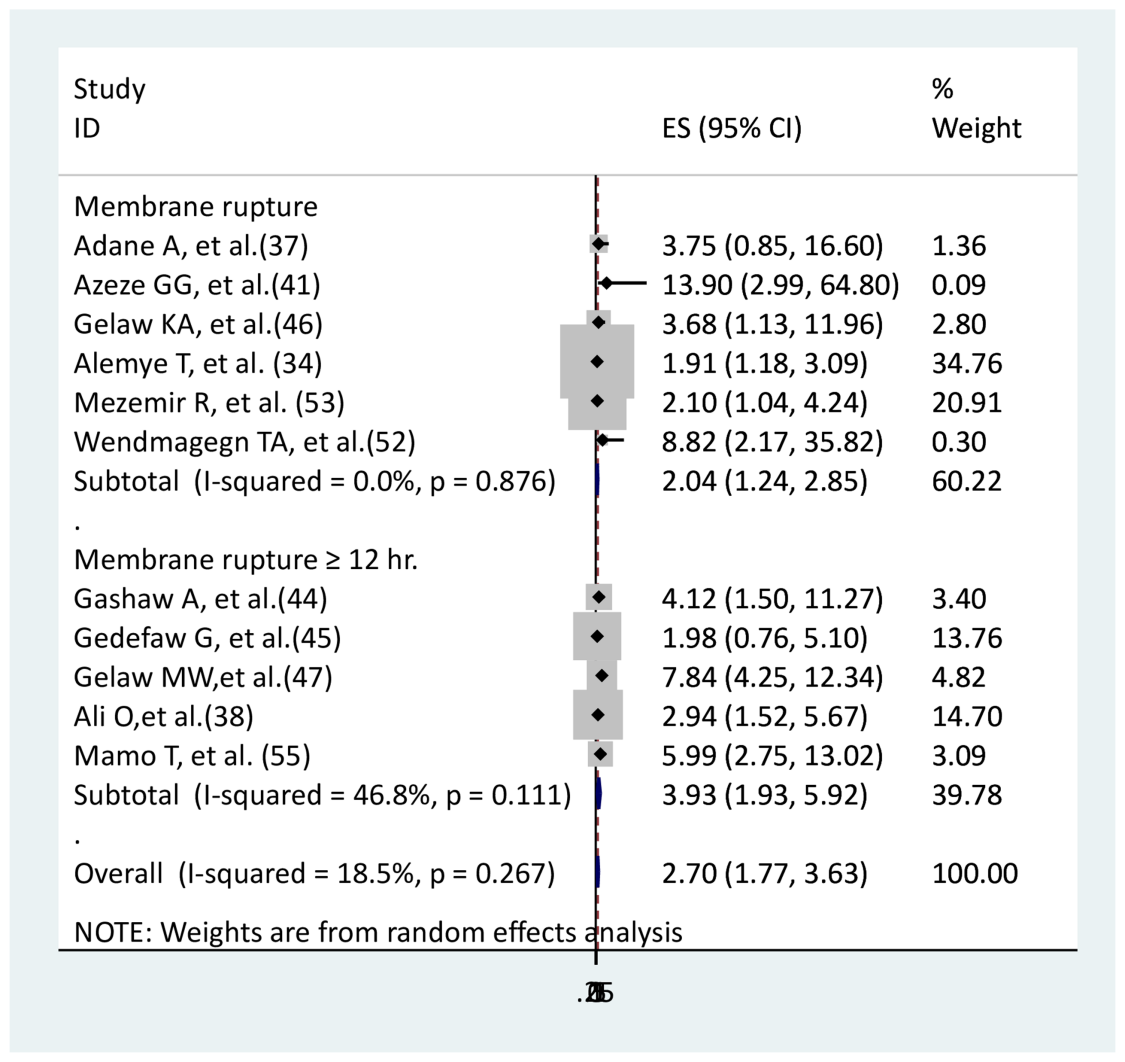
The association between membrane rupture and surgical site infections.

The relationship between membrane rupture and surgical site infection was examined in five studies (34, 37, 41, 46, 52, 53), while the relationship with membrane rupture greater than or equal to 12 h was examined in five studies (38, 44, 45, 47, 55). One study for mothers with membrane rupture (37) and the other study for those with membrane rupture greater than or equal to 12 h (45) did not show a significant association, while the remaining study had a significant association. Mothers with membrane ruptures (AOR = 2.04, 95% CI: 1.24–2.85), as well as those ruptured of membrane greater or equal to 12 h (AOR = 3.93, 95%CI: 1.93–5.92), were more likely to develop surgical site infection than mothers with intact membrane and ruptured membrane less than 12 h, respectively (Figure 9).

### The association between types of anesthesia and surgical site infections

**Figure 10:**
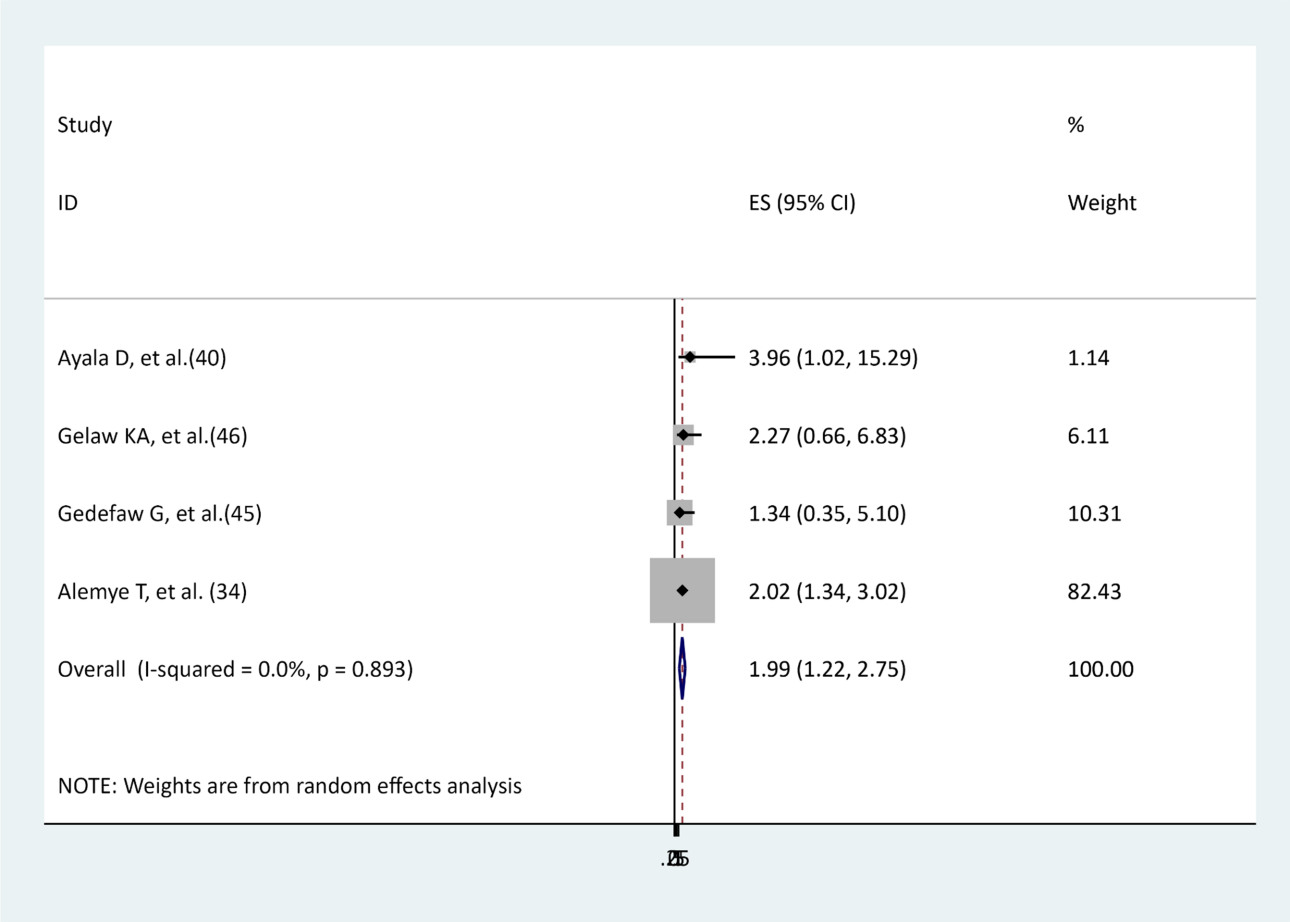
The association between types of anesthesia and surgical site infections.

To examine the effect of general anesthesia on surgical site infection, four studies were identified (34, 40, 45, 46). Half of the studies (34, 40) found a significant association between general anesthesia and surgical site infections; the other half (45, 46) did not detect such a significant association. The results of this meta-analysis showed that women who underwent general anesthesia during cesarean section were more likely to develop a surgical site infection than those who underwent spinal anesthesia (AOR = 1.99, 95% CI: 1.22–2.75), and there was no evidence of heterogeneity (I^2^ = 0, *p* = 0.893; Figure 10).

### The association between skin incision and surgical site infection

**Figure 11:**
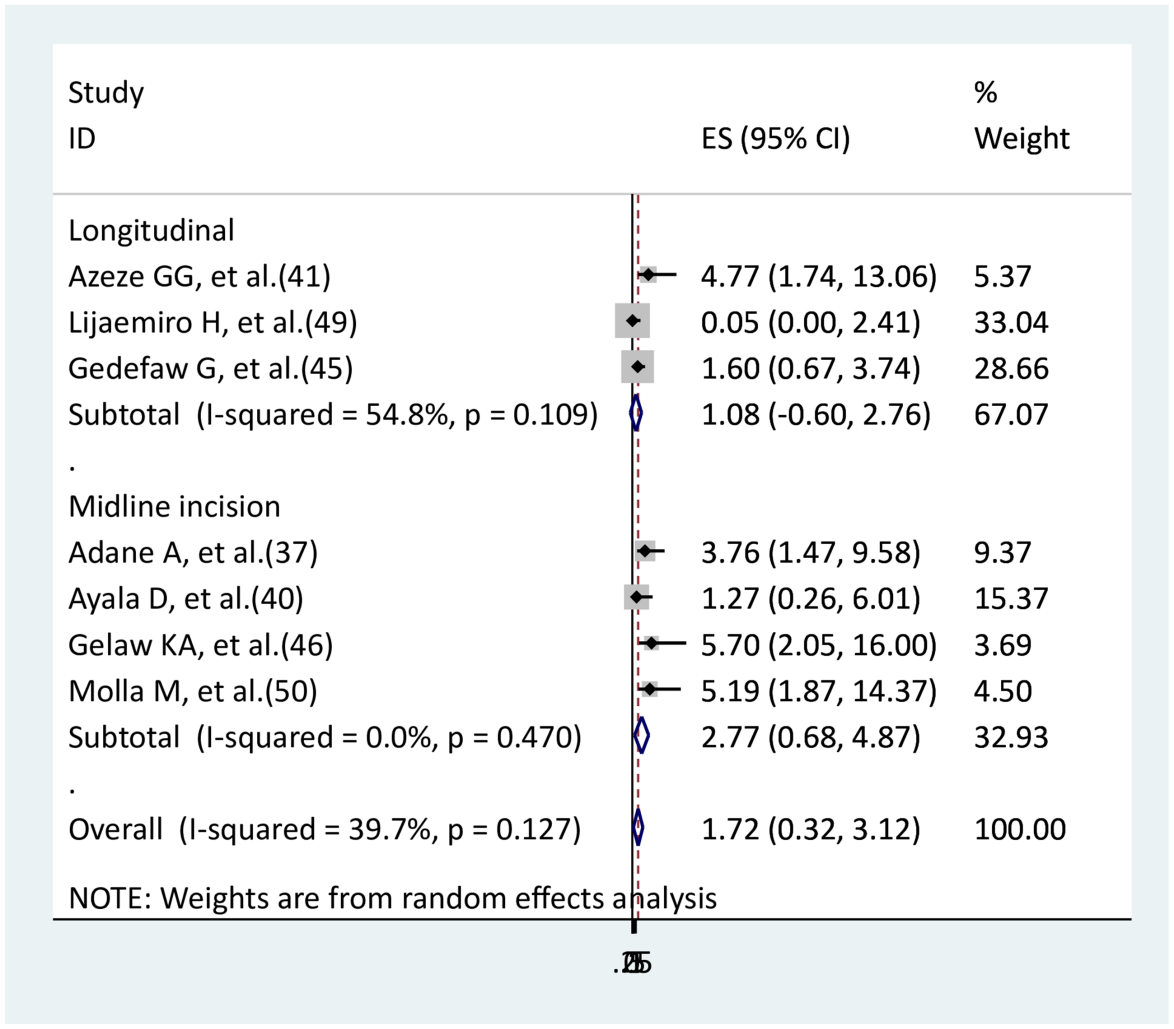
The association between skin incision and surgical site infection.

Three studies (41, 45, 49) were used to examine the relationship between longitudinal incision and surgical site infection, while four studies (37, 40, 46, 50) were used to examine the relationship between midline incision and an infection at the surgical site. In the longitudinal incision, only one study (41) had a significant association, whereas in the midline incision, only one study (40) did not have a significant association. The remaining had significant associations in both midline and longitudinal. In this meta-analysis, neither a midline (AOR = 2.77, 95% CI: 0.68–4.87) nor a longitudinal (AOR = 1.08, 95% CI: 0.6–2.76) skin incision showed a significant association with the surgical site in comparison to a low transverse skin incision (Figure 11).

### The association between skin closure and surgical site infection

**Figure 12:**
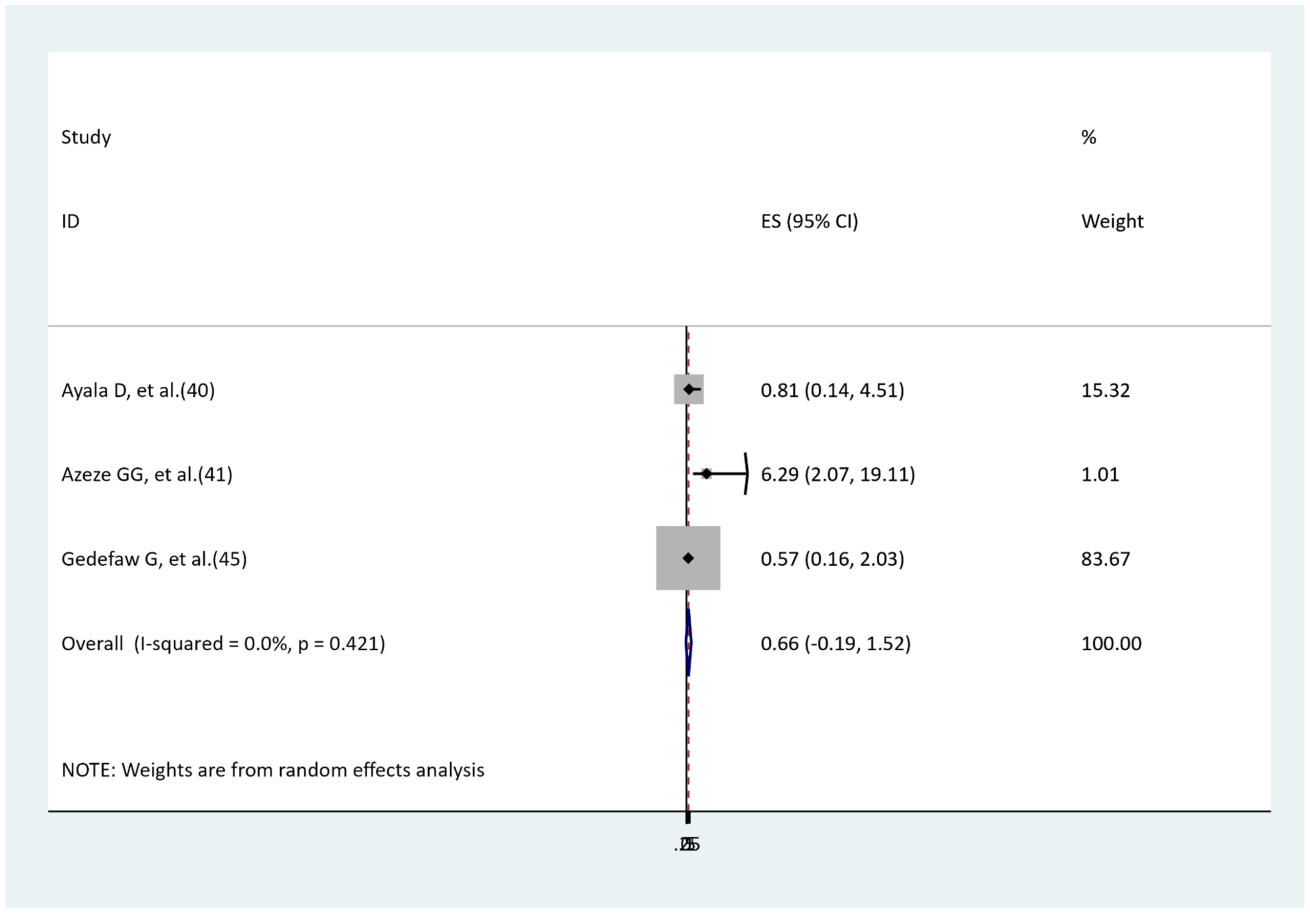
The association between skin closure and surgical site infection.

Three studies’ findings were reviewed to assess the relationship between skin closure and surgical site infection (40, 41, 45). One study’s findings indicated a significant relationship (41), whereas two studies’ findings showed no significant relationship (40, 45). The findings of the meta-analysis reported that no significant relationship was revealed between interrupted skin closure and surgical site infection as compared to subcuticular skin closure (AOR = 0.66, 95%: −0.19–1.52), with no heterogeneity (I^2^ = 0, *p* = 0.421; Figure 12).

### The association between the level of post-operative hemoglobin and surgical site infection

**Figure 13:**
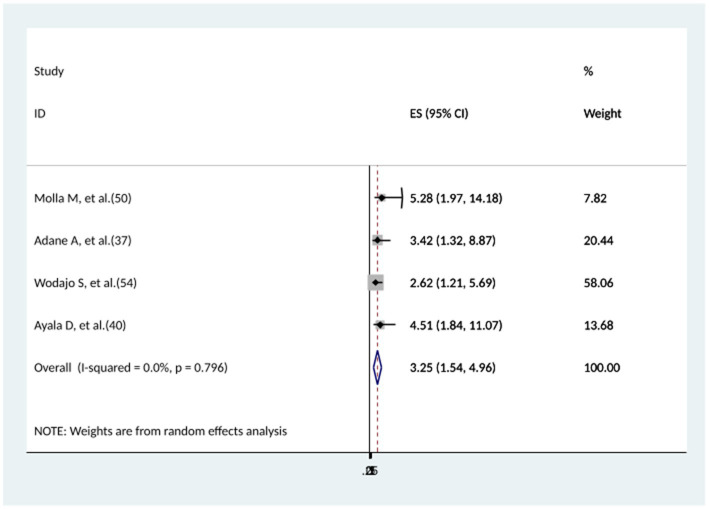
The association between the level of post-operative hemoglobin and surgical site infection.

The association between post-operative Hgb level and surgical site infection was determined by four studies (37, 40, 50, 54). All of the studies found a significant association. In this systematic review study, cesarean-delivered women who had post-operative Hgb levels less than 11 mg/dL during cesarean-section had a nearly 3 times higher risk of developing surgical site infection than women who had post-operative Hgb levels higher than 11 mg/dL (AOR = 3.25, 95% CI: 1.54–4.96), with the absence of heterogeneity (Figure 13).

## Discussion

Surgical site infection (SSI) is a major contributor to postoperative morbidity and mortality in developing countries. It is important to determine the magnitude of surgical site infection and its associated risk factors before, during, and after cesarean delivery in order to design prevention strategies in Ethiopia. In the current systematic review, the pooled incidence of surgical site infection was 12.32% (95% CI: 8.96–16.11%). This finding is slightly in line with a study done in Nepal (7). However, the current finding is significantly higher than studies conducted at the global level (4), in China (5), India (6), and the United Arab Emirate (8). The difference may be caused by socio-economic variation, poor dietary behaviors, poor personal hygiene, inadequate infection control practices implemented in healthcare, such as limited hygienic practice, inadequate antibiotic prophylaxis, a lack of aseptic wound care, and non-adherence with prescribed treatments. The other differences may be the sampling method of the study population and the study design. The current study’s estimate of surgical site infection is also somewhat consistent with the WHO report for middle- and low-income countries (9) and a study in Africa (10), but it is significantly higher than findings from studies conducted in sub-Saharan Africa (11) and Rwanda (12). The variation may be due to patient-related characteristics and inconsistent infection prevention strategies implemented across the countries. The pooled estimate of surgical site infection for this systematic review is higher than that of studies conducted in Ethiopia (14, 15), but it is nearly consistent with one study, which was 10.4% (13). The discrepancy could be attributed to factors relating to study participants, such as the patients’ own microbial flora (3), educational level, nutritional status, comorbidities, and sample size. In the subgroup analysis by region, the southern nation nationality of people (SNNP) reported the highest surgical site infection, followed by equivalent magnitudes in the Addis Ababa and Harari regions, and the Tigray region reported the lowest surgical site infection. This difference might be explained by regional socio-economic variation, inadequate postoperative care, poor infection control practice, and undertrained medical staff in Ethiopia. Therefore, regional contextual intervention, an appropriate infection prevention policy, and strict postoperative care should be implemented in the country. The incidence of surgical site infection was comparable in cohort and cross-sectional studies, as well as in the sample sizes of ≤422 and > 422. In the subgroup analysis by publication year, the incidence of surgical site infection was higher after 2020. As a result of the COVID-19 pandemic, infection prevention control for mothers during delivery and receiving post-partum care during this period may be quite challenging for staff, patients, and support personnel due to a relative lack of evidence-based practices, high rates of disease transmission, and shortages of personal protective equipment. In this meta-analysis, women living in rural areas were more likely to develop surgical site infections than women living in urban areas. This discrepancy could be explained by the microbial environment of rural women (3), the difficulties in treating urinary tract infections in pregnant women (21) due to the lack of access to health care facilities, and the common practice of female genital mutilation among rural women (20). Pregnant women who underwent general anesthesia had a higher risk of surgical site infections compared to pregnant women who got spinal anesthesia. This might be because pregnant women under general anesthesia spend a longer period in the hospital than those under spinal anesthesia, which increases the risk of nosocomial infections. The other cause may be that those pregnant women may be exhausted prior to a cesarean section by pregnancy-related complications such as antepartum hemorrhage, gestational diabetes, and premature rupture of the membrane, which could lead to an infection at the surgical site. In this study, mothers with ruptured membranes and chorioammionitis were more likely to develop surgical site infections than mothers with intact membranes. This evidence is consistent with the previous findings (18, 19). This might be because the membrane’s protective function in the cervical canal is lost once it is damaged. Since the sterile and protective membrane was ruptured and removed, every bacterial infection from a female genital tract infection (20) has a chance to ascend through the cervical aperture. This study also found that mothers who had a postoperative Hgb level below 11 mg/dL were more likely to get surgical site infections. These findings support previous findings (21, 22), which reported that postpartum hemorrhage and anemia were risk factors for surgical site infection. This might be a result of anemia depriving the wound of oxygen, which raises the risk of wound infection by impairing macrophage function and slowing the process of wound healing. Mothers who ruptured the membrane for more than or equal to 12 h were more likely to develop a surgical site infection than mothers who ruptured the membrane less than 12 h. The amniotic fluid surrounding the fetus begins to flow or spill out of the woman’s vagina as the membranes tear for a long time. This may be problematic since the absence of amniotic fluid raises the risk of infection, preterm birth, and other issues. In this meta-analysis, there is no difference in surgical site infections between elective and emergency cesarean sections. The Ethiopian government places high emphasis on maternal and child health, which includes cesarean sections. Pregnancy-labor medications and services can be covered by health insurance (cost of labor and delivery) in Ethiopia. This helps to reduce the discrepancy in infection prevention practices and controls, particularly for those unable to cover their medications. The infection prevention and control practices in any health institute are the same for elective and emergency cesarean sections. Due to these reasons, there may not be a significant difference in surgical site infection between emergency and elective cesarean sections. Longitudinal and midline incisions did not have a significant difference in surgical site infection compared to low transverse incisions. This may be due to the fact that, as noted, the confidence interval of longitudinal and midline incisions had a wider confidence interval, which indicates the sample size to detect the outcomes is inadequate.

One of the limitations of this study is that it did not include all regions of Ethiopia. The majority of studies included in this study were cross-sectional, which cannot establish a temporal relationship between surgical site infection and risk factors. This study’s other drawback is that it found large values of I^2^, which implies that there is heterogeneity among the studies. Even after running subgroup analyses, there was still significant heterogeneity among studies, demonstrating that the study variables explain almost everything. The presence of heterogeneity may result from variations among study participants and variations in study methodology.

## Conclusion

In this systematic review, more than one in 10 women delivered via cesarean section developed a surgical site infection. This may be caused by inadequate infection control practices in the nation’s healthcare system. Therefore, efforts to mitigate maternal mortality and morbidity must focus not only on expanding the quantity and accessibility of care but also on improving the quality of existing health care. Regional context intervention should be implemented. Furthermore, evidence-based care using a robust study design should be necessary considering the increased incidence of surgical site infection during the COVID-19 pandemic (since 2020). The Ethiopian Ministry of Health and its partners should place special attention on preventing rupture of membranes, chorioammionitis, and low postoperative Hgb levels, which would also have a useful synergistic effect on cesarean section site infection. Moreover, medical professionals would avoid using general anesthesia as a standard procedure for cesarean sections in order to reduce the risk of surgical site infection in women.

## Data availability statement

The original contributions presented in the study are included in the article/Supplementary material, further inquiries can be directed to the corresponding author.

## Author contributions

TGW: Conceptualization, Data curation, Formal analysis, Funding acquisition, Investigation, Methodology, Project administration, Resources, Software, Supervision, Validation, Visualization, Writing – original draft, Writing – review & editing. JAM: Conceptualization, Data curation, Formal analysis, Funding acquisition, Investigation, Methodology, Project administration, Resources, Software, Supervision, Validation, Visualization, Writing – original draft, Writing – review & editing.
