# Supplementary material for: The incidence of surgical site infection and its predictors among women delivered via cesarean sections in Ethiopia: a systematic review and meta-analysis
Source: Front Med (Lausanne). 2024 Apr 25;11:1395158. doi: 10.3389/fmed.2024.1395158 (PMC11079214; doi:10.3389/fmed.2024.1395158)
Supplement: Supplementary file 2 [file Table_2.DOCX]

S2 File. Comprehensive search strategy for the incidence of surgical site infection

| **Databases** | **Key search terms or phrases** | | | | | |
| --- | --- | --- | --- | --- | --- | --- |
| **PubMed** | ((((((((((((((((((("Surgical Wound Infection"[MeSH Terms]) OR ("Surgical Wound Infection")) OR ("Infections, Surgical Wound")) OR ("Surgical Wound Infections")) OR ("Wound Infections, Surgical")) OR ("Infection, Surgical Wound")) OR ("Surgical Site Infection")) OR ("Infection, Surgical Site")) OR ("Infections, Surgical Site")) OR ("Surgical Site Infections")) OR ("Wound Infection, Postoperative")) OR ("Wound Infection, Surgical")) OR ("Infection, Postoperative Wound")) OR ("Infections, Postoperative Wound")) OR ("Postoperative Wound Infections")) OR ("Wound Infections, Postoperative")) OR ("Postoperative Wound Infection")) OR ("surgical site infections")) AND ((((((((((((("Cesarean Section"[MeSH Terms]) OR ("Cesarean Section")) OR ("Cesarean Sections")) OR ("Delivery, Abdominal")) OR ("Abdominal Deliveries")) OR ("Deliveries, Abdominal")) OR ("Caesarean Section")) OR ("Caesarean Sections")) OR ("Abdominal Delivery")) OR ("C-Section (OB)")) OR ("C Section (OB)")) OR ("C-Sections (OB)")) OR ("Postcesarean Section"))) AND ((Ethiopia[MeSH Terms]) OR (Ethiopia)) | | | | | |
| Total articles | 23 | | | | | |
| **CINAHL** | | | “Surgical wound infection AllFields AND “Cesarean Section” AllFields AND “Ethiopia” AllFields | | | |
| Total articles | | | 63 | | | |
| **Google scholar** | | “Surgical wound infection” “Cesarean Section” “Ethiopia” | | | | |
| Total articles | | 133 | | | | |
| **African Journals Online** | | | | “surgical wound infection” “Cesarean Section” “Ethiopia” | | |
| Total articles | | | | 6 | | |
| **Ethiopian higher education institutional repositories** | | | | | | Surgical site infection among Cesarean Section women in Ethiopia |
| Total articles | | | | | | 2 |
| **Using reference list** | | | | Surgical site infection among Cesarean Section women in Ethiopia | | |
| Total articles | | | | 3 | | |
| **Total articles retrieved from all search strategies** | | | | | 230 | |
