# Supplementary material for: The incidence of surgical site infection and its predictors among women delivered via cesarean sections in Ethiopia: a systematic review and meta-analysis
Source: Front Med (Lausanne). 2024 Apr 25;11:1395158. doi: 10.3389/fmed.2024.1395158 (PMC11079214; doi:10.3389/fmed.2024.1395158)
Supplement: Supplementary file 3 [file Table_3.DOCX]

S3 File Data extraction format for the incidence of surgical site infections

| ID | Authors | Publication  year | Region | Study design | Sample size | Cases (prevalence) |  |
| --- | --- | --- | --- | --- | --- | --- | --- |
| 1. | Adane A, et al.(37) | 2022 | Harari | cross-section | 336 | 26 (7.74% |  |
| 2. | Alemye T, et al. (34) | 2021 | Harari | cross-section | 1069 | 131 (12.3%) |  |
| 3. | Ali A.(33) | 2017 | Amhara | cross-section | 166 | 12 (7.2%) |  |
| 4. | Ali O,et al.(38) | 2021 | Amhara | cross-section | 818 | 100 (12.2%) |  |
| 5. | Amenu D, et al.(39) | 2011 | Oromia | cohort | 580 | 66 (11.4%) |  |
| 6. | Ayala D, et al.(40) | 2021 | Oromia | cross-section | 382 | 34 (8.9%) |  |
| 7. | Azeze GG, et al.(41) | 2019 | Amhara | cross-section | 383 | 30 (7.8%) |  |
| 8. | Bizuayew H, et al.(42) | 2021 | Amhara | cross-section | 622 | 77 (12.4%) |  |
| 9. | Dach AM , et al.(43) | 2018 | SNNP | cross-section | 325 | 42 (12.9%) |  |
| 10. | Gashaw A, et al.(44) | 2022 | SNNP | cross-section | 431 | 51 (11.8%) |  |
| 11. | Gedefaw G, et al.(45) | 2018 | Amhara | cross-section | 447 | 42 (9.4%) |  |
| 12. | Gelaw KA, et al.(46) | 2017 | Tigray | cross-section | 384 | 26 (6.8%) |  |
| 13. | Gelaw MW,et al.(47) | 2018 | Addis Ababa | cross-section | 474 | 40 (8.4%) |  |
| 14. | Kebede A.(35) | 2022 | SNNP | cross-section | 226 | 179 (79.2%) |  |
| 15. | Ketema DB, et al.(48) | 2020 | Amhara | cohort | 520 | 132 (25.4%) |  |
| 16. | Lijaemiro H, et al.(49) | 2020 | Addis Ababa | cohort | 166 | 25 (15%) |  |
| 17. | Molla M, et al.(50) | 2019 | Amhara | cross-section | 334 | 27 (8.1%) |  |
| 18. | Rose AF, et al.(51) | 2018 | Amhara | cohort | 247 | 21 (8.6%) |  |
| 19. | Wendmagegn TA, et al.(52) | 2018 | Tigray | cross-section | 206 | 24 (11.7%) |  |
| 20. | Negese K, et al (36). | 2023 | Amhara | cross-section | 368 | 9 (2.4%) |  |
| 21 | Mezemir R, et al. (53) | 2023 | Addis Ababa | cohort | 741 | 86 (11.6%) |  |
| 22 | Wodajo S, et al. (54) | 2017 | SNNP | cross-section | 592 | 65 (11%) |  |
| 23 | Mamo T, et al. (55) | 2017 | Oromia | cross-section | 384 | 36 (9.4%) |  |

SNNP = Southern Nation Nationality People. Dash (-) indicates no percentage reported.
