# Supplementary material for: The incidence of surgical site infection and its predictors among women delivered via cesarean sections in Ethiopia: a systematic review and meta-analysis
Source: Front Med (Lausanne). 2024 Apr 25;11:1395158. doi: 10.3389/fmed.2024.1395158 (PMC11079214; doi:10.3389/fmed.2024.1395158)
Supplement: Supplementary file 4 [file Table_4.DOCX]

|  | | S4 File. Displays the results of the quality assessment of the included studies after being generally agreed upon by the two authors (TGW and JAM) | | | | | | | | | | | | | | | | |  | |  |
| --- | --- | --- | --- | --- | --- | --- | --- | --- | --- | --- | --- | --- | --- | --- | --- | --- | --- | --- | --- | --- | --- |
| **id** | JBI Critical Appraisal Checklist for cross-sectional studies | | Agreed-upon results | | | | | | | | | | | | | | | | | |  |
|  | **References** | | **35** | **46** | **34** | **33** | **38** | **40** | **41** | **42** | **43** | **44** | **45** | **47** | **50** | **52** | **36** | **54** | | **55** |  |
| 1 | Were the criteria for inclusion in the sample clearly defined? | | y | y | y | y | n | y | y | n | n | n | y | y | y | n | y | y | | y |  |
| 2 | Were the study subjects and the setting described in detail? | | y | y | y | y | y | y | y | y | y | y | y | y | y | y | y | y | | y |  |
| 3 | Was the exposure measured in a valid and reliable way? | | y | y | y | y | y | y | y | y | y | y | y | y | y | y | y | y | | y |  |
| 4 | Were objective, standard criteria used for measurement of the condition? | | y | y | y | y | y | y | y | y | y | y | y | y | y | y | y | y | | y |  |
| 5 | Did confounding factors identify? | | y | y | y | y | y | y | y | y | y | y | y | y | y | y | n | y | | y |  |
| 6 | Were strategies to deal with confounding factors stated? | | y | y | y | y | y | y | y | y | y | y | y | y | y | y | n | y | | y |  |
| 7 | Were the outcomes measured in a valid and reliable way? | | y | y | y | y | y | y | n | y | n | n | y | y | y | y | y | y | | y |  |
| 8 | Was appropriate statistical analysis used? | | y | y | y | y | y | y | y | y | y | y | y | y | y | y | y | y | | y |  |
| Score | | | 8/8 | 8/8 | 8/8 | 8/8 | 7/8 | 8/8 | 7/8 | 7/8 | 6/8 | 6/8 | 8/8 | 8/8 | 8/8 | 7/8 | 6/8 | 8/8 | | 8/8 |  |
| Overall appraisal percent (%) | | | 100% | 100% | 100% | 100% | 87.5% | 100% | 87.5% | 87.5% | 75% | 75% | 100% | 100% | 100% | 87.5% | 75% | 100% | | 100% |  |

| No. | JBI Critical Appraisal Checklist for cohort studies | Agreed-upon results | | | | | |
| --- | --- | --- | --- | --- | --- | --- | --- |
|  | **References** | **37** | **39** | **48** | **49** | **51** | **53** |
| 1 | Were the two groups similar and recruited from the same population? | y | y | y | y | y | y |
| 2 | Were the exposures measured similarly to assign people to both exposed and unexposed groups? | y | y | y | y | y | y |
| 3 | Was the exposure measured in a valid and reliable way? | y | y | y | y | y | y |
| 4 | Were confounding factors identified? | y | n | y | y | y | y |
| 5 | Were strategies to deal with confounding factors stated? | y | n | y | y | y | y |
| 6 | Were the groups/participants free of the outcome at the start of the study (or at the moment of exposure)? | y | y | y | y | y | y |
| 7 | Were the outcomes measured in a valid and reliable way? | y | y | y | n | y | y |
| 8 | Was the follow up time reported and sufficient to be long enough for outcomes to occur? | y | y | y | y | NA | y |
| 9 | Was follow up complete, and if not, were the reasons to loss to follow up described and explored? | y | y | y | y | NA | y |
| 10 | Were strategies to address incomplete follow up utilized? | y | y | y | y | NA | y |
| 11 | Was appropriate statistical analysis used? | y | n | y | y | y | y |
| Score | | 11/11 | 8/11 | 11/11 | 10/11 | 8/8 | 11/11 |
| Overall appraisal percent (%) | | 100% | 72.7% | 100% | 90.9% | 100% | 100% |

**y=yes, n=no, NA= not applicable. Scores of 50% and above were considered low-risk, while those below 50% were considered high-risk.**
